# Sexual Health Outcomes of Adolescent and Young Adult Colorectal Cancer Survivors and Their Partners: Protocol of a Dyadic Mixed Methods Study

**DOI:** 10.2196/41831

**Published:** 2023-03-23

**Authors:** Chiara Acquati, Daniela Wittmann, Michael Roth, Allison Rosen, Lynley Christian Carr, Zachary Gresham, Elena Ionescu

**Affiliations:** 1 Graduate College of Social Work University of Houston Houston, TX United States; 2 Department of Urology University of Michigan Ann Arbor, MI United States; 3 Department of Pediatrics Patient Care Division of Pediatrics The University of Texas MD Anderson Cancer Center Houston, TX United States; 4 American Cancer Society Kennesaw Georgia

**Keywords:** adolescent and young adult, colorectal cancer, oncology, cancer, couples, dyadic research, dyad, sexual health, sexual dysfunction, reproductive health, infertility-related distress, mixed methods design, mixed method, adolescent, adolescence, young adult, infertility, fertility

## Abstract

**Background:**

Sexual dysfunction represents a critical aspect of quality of life for adolescent and young adult cancer survivors. Studies have consistently documented that adolescents and young adults report greater psychological and physical morbidity than older survivors and healthy peers, including elevated rates of sexual dysfunction, often accompanied by lower satisfaction with sex life and delays in meeting sexual milestones. Moreover, sexual dysfunction, body image concerns, and fertility status affect their confidence in being both physically and emotionally intimate. Despite this evidence, limited research has investigated the influence of psychosocial and interpersonal factors on sexual health outcomes reported by this group. This constitutes a significant gap in the provision of comprehensive sexual health care for adolescent- and young adult–onset cancer survivors, especially since greater emphasis has been recently placed on the biopsychosocial model of sexuality and dyadic approaches to intervention and treatment. In comparison to other cancer types, the incidence of colorectal cancer (CRC) has been increasing at an alarming rate for the adolescent and young adult group. Patients with early-onset CRC experience elevated rates of sexual dysfunction, psychological distress, and social and physical burden, often resulting from issues with bowel control, incontinence, and body image.

**Objective:**

This study uses an explanatory sequential mixed methods approach to (1) characterize sexual function, sexual distress, dyadic coping, infertility-related distress, relationship and mental health outcomes of adolescent and young adult CRC survivors within the first 5 years post diagnosis and their partners; (2) examine the reciprocal influence of sexual function and dyadic coping behaviors on sexual distress; and (3) identify interpersonal or couple characteristics associated with coping with sexual dysfunction and its associated distress.

**Methods:**

Participating couples (n=60) will complete a quantitative web-based survey investigating sexual function, sexual distress, dyadic coping, infertility-related distress, emotional functioning, relationship satisfaction, and body image (cancer survivors only). A subset of 20 couples will participate in an in-depth dyadic interview with 2 members of the research team to further explore couple-based strategies implemented to cope with cancer-related sexual dysfunction and distress.

**Results:**

The study received institutional review board approval. Recruitment and enrollment of couples began in July 2022.

**Conclusions:**

Results will provide a deeper understanding of the challenges couples experience as they navigate sexual intimacy after CRC treatment by highlighting the role of interpersonal processes. These findings will inform a dyadic intervention for young couples at risk of greater sexual distress in the aftermath of CRC.

**International Registered Report Identifier (IRRID):**

DERR1-10.2196/41831

## Introduction

### Background

Cancer-related sexual dysfunction is one of the most common and distressing consequences of the illness, which, if untreated, persists or worsens over time [1-4]. Among adolescents and young adults—individuals aged between 15 and 39 years at diagnosis—a considerable body of research has unveiled unique challenges [5,6] including disruption of normative physical, psychosocial, and emotional development, which can lead to sexual dysfunction [7-13]. Studies have consistently documented that adolescents and young adults report greater psychological [14,15] and physical morbidity [5,6,16] than older survivors and healthy peers, including elevated rates of sexual dysfunction [11,13,17-21] often accompanied by lower satisfaction with sex life [22-26], delays in meeting sexual milestones [21,27,28], and reduced frequency of sexual activity [21,27]. Between 42% and 52% of adolescent and young adult survivors experience 1 or more sexual problems [12,13,29], with one-third of them reporting worse sexual function up to 2 years post diagnosis [13]. Differences exist by sex, with females reporting higher dysfunction scores [30-32]. Young female survivors experience greater problems with pain, lubrication, desire, and sexual satisfaction than healthy peers [22-26,28]. Qualitative studies document that women report pain, lack of desire, and reduced sexual enjoyment, while males experience erectile dysfunction, ejaculation, and arousal problems [12]. While aggregate data from large studies report that the majority of adolescents and young adult survivors were involved in a relationship [12,21], cancer and treatment-related side effects proved to be detrimental to masculinity and perceived closeness with partners [21-27]. Sexual dysfunction, body image concerns, and fertility status may limit the confidence in being both physically and emotionally intimate [29,31,33,34] and contribute to more negative outcomes [29,31,33-35]**.**

In comparison with other cancer types, the incidence of colorectal cancer (CRC) has been increasing at an alarming rate [36-41] for the adolescent and young adult group. Recent reports have projected that by 2030, the largest increase in the incidence of colon (90%) and rectal (125%) cancer will occur for the 20-34–year-old group [36-39,42-45]. Treatments include a combination of chemotherapy, pelvic radiation, and abdominoperineal resection—often with a temporary or permanent ostomy—which negatively impact psychological, sexual, and reproductive health outcomes [46-49]. Patients with early-onset CRC experience elevated psychological distress and social and physical burden, often resulting from issues with bowel control or incontinence and body image [50,51]. Rates of sexual dysfunction are higher than those of other cancer types [51]. Additionally, approximately 48% of young patients with CRC reported that sexual health issues have negatively affected the relationship with their partner [52,53]. Despite the burden associated with the long-term symptoms that accompany this cancer type and elevated rates of impaired functioning, sexual health has been mostly addressed as an individual experience without exploring how young patients with and survivors of CRC cope with sexual distress in the context of their relationship with a partner [10,14,35,54,55].

It is well recognized that the multidimensional distress of cancer affects both patients and partners [3,56-59]. Yet, only a few studies have investigated sexual health outcomes from a couple perspective among adolescents and young adults despite 3 important considerations. First, partners play a critical role in providing emotional, physical, and practical support [56,57,59,60]. Intimate partners also contribute to patients’ adjustment to the illness: distress experienced by either partner has been found to directly affect distress levels in the other, with negative outcomes in quality of life, relationship functioning, and mental health for discordant couples [61-64]. Second, integrative models of sexuality place greater emphasis on psychosocial and interpersonal factors [65,66]. This finding supports the notion that greater attention should be placed on the psychosocial experience of both members of the dyad, in terms of shared loss and coordinated strategies to cope with cancer-related sexual dysfunction [3,60,67,68]. Finally, recent findings indicate that interventions addressing both partners’ experiences in the context of sexual recovery and treatment are feasible and acceptable and they improve outcomes for both [58,69-76]. Hence, there is a critical need to investigate interpersonal characteristics that influence adolescent and young adult cancer survivors and their partners’ experience with sexual dysfunction. This knowledge gap affects the provision of comprehensive sexual health care for adolescent and young adult cancer survivors [12,19,20,35] and the development of interventions tailored to the needs of individuals and couples at higher risk of sexual distress during survivorship.

This paper describes the protocol of the AYA CRC SHARE (ie, Sexual Health And REproductive concerns) project, which investigates sexual and reproductive issues of young adult CRC survivors and their partners within the first 5 years after diagnosis. This study will fill a significant gap in the current body of evidence assessing the long-term consequences of treatments for CRC on sexual health and wellbeing–related outcomes. In addition, this investigation will inform a subsequent intervention to enhance couples’ coping and communication skills critical to sexual intimacy.

### Theoretical Model

This study is underpinned by a theoretical model that integrates the Biopsychosocial Model of Sexuality after Cancer developed by Bober and Varela [65], with Wittmann’s Sexual Recovery Model [67,77,78]. Bober and Varela [65] have outlined an integrative biopsychosocial model, where cancer-related sexual problems exist at the intersection of biological (ie, body image change, fatigue, pain, and loss of sensation), psychological (ie, depression and anxiety), sociocultural (ie, social and cultural norms), and interpersonal factors such as relationship quality, intimacy, and communication [65]. Wittman [74,78] has extended this work, illustrating how changes in sexual functioning after cancer represent a loss for both patients and partners. Their model conceptualizes sexual recovery as a process that shares similarities with the grief experience [67,74,77-79]. Finally, if we consider cancer and the resulting sexual loss as a dyadic stressor, it is possible to postulate that dyadic coping strategies are needed to adjust to this transition. According to the Systemic-Transactional Model [80], dyadic coping includes both partners’ strategies to manage their own and their partners’ distress as well as coordinated coping behaviors implemented by the dyad in response to stressful situations that may affect the quality of the relationship. Therefore, our work investigates whether dyadic coping behaviors that characterize the exchange within young partners may alleviate the burden associated with sexual distress experienced after cancer.

## Methods

### Study Design

**Figure 1:**
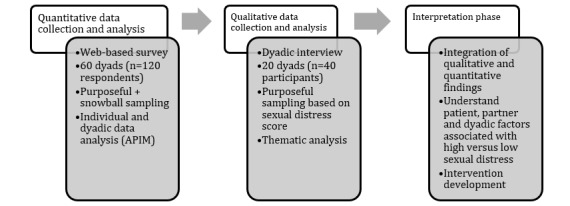
Overview of the mixed methods explanatory sequential design of the study. APIM: Actor-Partner Interdependence Model.

This study will use an explanatory sequential mixed methods approach [81] to (1) characterize sexual function, sexual distress, body image, fertility concerns, and dyadic coping among adolescent and young adult CRC survivors and their partners using validated measures (n=60 couples); (2) examine the reciprocal influence of each partner’s sexual function (model 1) and dyadic coping style (model 2) on self-reported sexual distress; (3) identify interpersonal or couple characteristics associated with their ability to cope with sexual dysfunction; and (4) integrate quantitative and qualitative findings to expand the current understanding of dyadic adjustment to sexual distress (Figure 1).

### Inclusion and Exclusion Criteria

The target sample includes 60 dyads composed of young adult CRC survivors and their romantic partners (n=120). A subset of 20 couples (n=40) will complete an in-depth dyadic interview with 2 members of the research team to further explore couple-based strategies implemented to cope with cancer-related sexual dysfunction. Couples will be selected for the qualitative component of the study based on their score on the sexual distress measure (see *Quantitative Web-Based Survey Study Component*). Couples scoring in the highest and lowest 10th percentile will be invited to complete qualitative interviews. Inclusion criteria for cancer survivors consist of (1) having received a diagnosis of CRC (stages I-IV) within 5 years prior to study enrollment; (2) being in a romantic relationship with a partner; (3) being older than 18 and younger than 39 years at the time of diagnosis; and (4) speaking English. Intimate partners are included in the study if they are (1) older than 18 years and (2) able to speak in English. Exclusion criteria are (1) cognitive impairment or severe mental illness that would impede the completion of self-report instruments; (2) attending couples therapy at the time of survey completion; (3) currently receiving end-of-life care; and (4) the partner is not present or unwilling to participate. No exclusions will be made on the basis of gender identity, sexual orientation, ethnicity, or cultural background.

### Participant Recruitment and Study Procedure

Multiple recruitment strategies will be implemented to reach the expected sample size of adolescent and young adult couples inclusive of CRC survivors and their partners. Informational flyers and social media scripts will be distributed among advocacy and community-based organizations for individuals with CRC, adolescents and young adults with cancer, and sexual or reproductive health with the help of a CRC survivor and advocate who is a member of the research team (AR). Investigators will also recruit participants at CRC events and conferences. We will work closely with the University of Texas MD Anderson Cancer Center Adolescent and Young Adult Program to promote study recruitment through existing listerves and private social media groups. In addition to active recruitment, study investigators (CA, DW, and MR) will share the study with psychosocial oncology providers and pediatric or medical oncologists to encourage them to refer participants to the study.

Potential participants can self-refer to the study in two ways: (1) by clicking on the URL and QR code, which will link them to a web-based study information form and screening questions or (2) by contacting the research team directly via email or telephone. The study information form includes a list of exclusionary criteria, and potential participants can opt out of the study if they present any of the characteristics that would interfere with their ability to qualify for the study activities. To preserve the integrity of the data, a brief telephone screening ascertaining age, cancer diagnosis, and relationship status will be implemented. Upon successful recruitment, participants will receive an individualized link to complete the web-based survey. They will be asked for their permission for the study team to contact their romantic partners, and couple completion of the survey will be monitored through individual and dyadic identifiers. Inclusion in the study is based on both partners’ provision of informed consent and completion of study questionnaires. If only one partner completes the study questionnaire or if less than 50% of the survey questions are answered, they will be excluded from the final sample. Two reminders will be sent to the participants and their partners. Additionally, among couples who express interest in participating in the qualitative component of the study while completing the survey, dyadic interviews will be conducted, with 10 couples reporting elevated sexual distress and 10 couples reporting low sexual distress (based on our sample distribution of scores) to appraise strategies implemented to cope with cancer-related sexual dysfunction.

### Ethical Considerations

This study will be performed in accordance with the tenets of the Declaration of Helsinki. The University of Houston’s institutional review board has reviewed and approved the study protocol (reference number STUDY00003538). Informed consent will be obtained from each participant before commencement of study research activities.

Recruitment and consenting of study participants will occur mostly through web- and internet-based approaches. A cover letter precedes the web-based survey explaining the study aims and procedures. Written information describing the research will be provided to survey participants, and this information will reference the optional interview procedures for a portion of the sample. Upon survey completion, a US $30 Amazon gift card (US $60 per couple) will be distributed via email. Additionally, a US $60 electronic gift card will be provided to each partner after participating in the dyadic interview (US $120 per couple).

Although this is considered a low-risk study, it is possible that answering questions about cancer diagnosis, sexual and reproductive health, as well as cancer-related impact on the couple’s relationship may cause participants to experience distress, sadness, or anxiety. The study team will provide participants with resources for cancer-related support in the follow-up thank you message used to disseminate the incentive. Participants can also contact the investigators or let the study team members know about their interest in being referred to community-based organizations. For partners who will participate in the qualitative interview, potential aspects of distress include cancer-related distress, sexual, fertility, and negative relational outcomes. In addition, mental health and suicide risk may emerge in the exchange with the investigators. Both participants (survivor and healthy partner) will receive a copy of the resource list created ad hoc from the research team, which includes the National Suicide Prevention Lifeline link and a 24/7 phone number. Being in an electronic format, the resource list will be attached to the thank you email for the participants, and it can also be shown directly to them upon conclusion of the interview. Other risks include breach of confidentiality or privacy. Efforts will be made to limit the potential for privacy to be threatened by a data breach: first, we made the decision of not pairing data from the web-based survey and the interview with identifying information. When data are downloaded, they will be stored in a password-protected encrypted computer. Each subject’s name will be paired with a code number, which will appear on written study material. The list pairing the subject’s name to the assigned code number will be kept separate from these materials, and only the study principal investigator will have access to this information.

### Participant Safety

Participant safety will be addressed through the implementation of the following strategies. First, survey instruments and study procedures will be reviewed and tested by expert e-patients and young CRC advocates [82] until consensus is achieved. Second, investigators will closely monitor responses to instruments assessing emotional functioning. An ad hoc list of resources in the areas of sexual health, mental health, caregiving, financial toxicity, and oncofertility has been compiled in collaboration with content experts or patient advocates, and it will be distributed to all participants at the end of the study activities. Third, 2 facilitators with expertise in research on sensitive health topics will conduct the interviews to reduce potential harm to participants because of the distress that may be elicited by the topics discussed [83], while affirming the cathartic and empowering aspect of using one’s experience to improve quality of care for adolescent and young adult couples. Additionally, to protect the individuals’ well-being and couples’ functioning, participants may opt to conduct the interview separately.

### Study Measures

#### Quantitative Web-Based Survey Study Component

Survivors and partners will be asked to complete questions about sociodemographic information including current age, sex, gender identity, sexual orientation, race and ethnicity, education, occupational status, insurance status, and income. Relationship characteristics include marital status, length of relationship, cohabitation, and presence of children. Clinical information for adolescent and young adult survivors will include cancer diagnosis, stage of the disease, age at diagnosis, time since diagnosis, and treatment type. Table 1 provides an overview of the sexual and psychosocial measures administered to participants.

Sexual function and satisfaction will be assessed with the Patient-Reported Outcomes Measurement Information System (PROMIS) Sexual Function and Satisfaction Measure version 2.0 Brief Profile (female version: 14 items, and male version: 10 items) [84,85]. The following domains will be answered by females: vaginal lubrication, vaginal pain, vulvar discomfort, and clitoral discomfort. The erectile function domain will be completed by male partners and survivors. The brief measure also includes 4 gender-neutral domains: interest in sexual activity, satisfaction with sex life, orgasm ability, and orgasm pleasure. All respondents are invited to complete items related to interest in sexual activity, whereas the remaining domains are to be completed only by respondents who had been sexually active. Per PROMIS network instructions, domain scores will be transformed to a *t* score distribution, where 50 represents the mean for the general US population [24,86]. A cutoff of 1 SD from the mean *t* score distribution of each domain will be used to define sexual dysfunction. The Sexual Function and Satisfaction Measure has shown adequate construct, content, known-groups validity, and test-retest reliability in adults and in groups of adolescents and young adults with cancer [84,85,87]. Because of the focus on young CRC survivors, 2 additional items will explore discomfort in the anus and rectum areas. Participants who do not identify as male or female in their sexual relationships are given the option to complete the General Measure of Sexual Satisfaction [88]. The instrument contains 5 semantic differential items rated on a 7-point scale. The total score, calculated as the sum of each item, ranges from 5 to 35 and higher values indicate greater sexual satisfaction. The instrument has demonstrated strong reliability and validity [88].

**Table 1:** Overview of the sexual and psychosocial measures included in the web-based survey.

Instruments		Items, n	Psychometrics
**Sexual function and satisfaction**			
	Patient-Reported Outcomes Measurement Information System Sexual Function and Satisfaction Measure Brief Profile	10 for males and 14 for females	Females: α=.89-.95; males: α=.71-.87
	Global Measure of Sexual Satisfaction	5	Cronbach α=.96
**Sexual distress**			
	Sexual and Relationship Distress Scale	30	Cronbach α=.95
**Dyadic coping**			
	Dyadic Coping Inventory	37	.68≥Cronbach α=≥.95 for subscales and total score
**Perceived infertility-related distress**			
	Fertility Problem Inventory (Relationship Concern and Need for Parenthood subscales)	10	Relationship Concern: α=.82; Need for Parenthood: α=.84
**Emotional functioning**			
	Patient Health Questionnaire-8	8	Cronbach α=.82
	Emotion Thermometers	5	Cronbach α=.90
**Relationship satisfaction**			
	General Measure of Relationship Satisfaction	5	Cronbach α=.96
**Body image**			
	Body Image Scale^a^	10	Cronbach α=.93

^a^Only for colorectal cancer survivors. This scale is not administered to partners.

Sexual distress will be measured with the Sexual and Relationship Distress Scale [89], which consists of 30 items assessing multidimensional (14-factor) individual and relationship distress experienced in the context of sexual difficulties. Each subscale score is calculated by combining the scores on each item, which ultimately determines the resulting total score. The instrument has demonstrated high internal consistency and high convergent and discriminant validity [89], and it has been administered in the context of breast and gynecological cancer survivorship in young adults [90].

Dyadic coping will be assessed using the Dyadic Coping Inventory [71,91-95], a 37-item questionnaire evaluating dyadic coping strategies implemented to cope with the stress of cancer on a 5-point Likert scale from 1=“very rarely” to 5=“very often.” Dyadic coping describes partners’ strategies implemented to manage their own and their partners’ distress as well as shared coping behaviors enacted by the dyad in response to stressful situations that affect the relationship. Items are organized in multiple subscales: stress communication, supportive, delegate, negative, and common dyadic coping. In addition, respondents are invited to rate their satisfaction with and the effectiveness of the dyadic coping behaviors implemented. Satisfactory psychometrics have been previously reported both for the original version of the instrument [94,95] and the English validated questionnaire [91,96].

Perceived infertility-related distress in cancer survivorship will be measured by administering the Relationship Concern and Need for Parenthood subscales (10 items each) from the Fertility Problem Inventory [97]. Each subscale and the instrument’s total score have demonstrated appropriate internal consistency and convergent and discriminant validity, with scores on the selected subscales being associated with marital adjustment. Current treatment protocols for CRC have long-term implications for fertility status [98-102], which can represent an additional source of distress for patients and their partners. Participants can opt out of these questions if they have completed their family-building or if the questions are considered not applicable.

Emotional functioning will be assessed with the Patient Health Questionnaire-8 [103,104] and the Emotions Thermometer [105,106]. The Patient Health Questionnaire-8 is a brief measure of depression validated both for the general population and for individuals with cancer [103,104]. Participants rate on a 4-point Likert scale that ranges from 0 to 3 (0=“not at all,” 1=“several days,” 2=“more than half of the days,” and 3=“nearly every day”) symptoms of depression experienced over the past 2 weeks; for example, feeling tired or having little energy, poor appetite, or overeating. The total score, obtained as a sum of each item, ranges from 0 to 24, with higher scores representing mild, moderate, and severe levels of depression. Excellent psychometric characteristics have been recorded across age and racial and ethnic groups, supporting its application in the context of psychosocial oncology [107-110]. The Emotions Thermometer is a self-report visual analogue tool including 5 domains (distress, anxiety, depression, anger, and need for help) on a scale ranging from 0 to 10. Excellent reliability (Cronbach α=.90), sensitivity (82.4%), and specificity (68.6%) of the instrument have been confirmed in multiple samples [105,106].

Relationship satisfaction will be measured using the General Measure of Relationship Satisfaction to examine the quality of the couple’s relationship. This 5-item scale contains five 7-point semantic differentials—“good-bad,” “pleasant-unpleasant,” “positive-negative,” “satisfying-unsatisfying,” and “valuable-worthless”—in response to the question, “How would you describe your relationship with your partner?” The scale has shown strong reliability and validity [88] in men (Cronbach α=.96) and women (Cronbach α=.96).

Body image concerns of CRC survivors will be assessed with the Body Image Scale [111,112]. Items assess how patients feel about their appearance and any changes that may have resulted from their disease or treatment (“Have you felt less physically attractive as a result of your disease or treatment?” and “Did you find it difficult to look at yourself naked?”). The 10 items are rated on 4-point response option consisting of “not at all,” “a little,” “quite a bit,” and “very much.” Higher scores are indicative of lower body image perception. The scale has demonstrated high reliability (Cronbach α=.93), discriminant validity, and sensitivity to change [111].

#### Qualitative Study Component

A subset of 20 couples (n=40) will complete an in-depth dyadic interview with 2 members of the research team to further explore strategies implemented by the 2 partners to cope with cancer-related sexual dysfunction and its associated distress. Dyads will be selected for the qualitative section of the study based on their sexual distress score (Sexual and Relationship Distress Scale [83]). Ten couples scoring in the highest and lowest 10th percentile of sexual distress will complete the qualitative interviews. Questions will address participants’ experience with sexual dysfunction, sources of sexual distress or concerns; perceived changes and consequences on couples’ relationships; and preferences for interventions addressing sexual health issues for patients with CRC and their partners. Couples will be contacted by the research team via email or telephone. Members of the research team will share information about the topics covered in the interviews, how interviews will be conducted, and how data will be collected, stored, and analyzed. Interested participants will be verbally consented at the beginning of the interview, which will be conducted either via telephone or via a Health Insurance Portability and Accountability Act–compliant web-based videoconferencing platform.

### Statistical Analysis

Univariate descriptive statistics and graphs will be analyzed to assess variables’ distributions. A missing data analysis will be conducted to determine whether list-wise deletion or multiple imputation will be appropriate; this will consider the amount of missingness and viability of the missing at random assumption necessary for multiple imputation [113]. Pearson *r* correlation coefficients will be used to assess the linear relationship among sociodemographic, clinical, and sexual or psychosocial measures, with specific attention to the variables of interest for the study. Comparisons of demographic characteristics between adolescent and young adult CRC survivors and their partners will be conducted using paired samples *t* tests for continuous variables and chi-square tests of association for nominal variables. Correction for multiple tests will be applied to control type I errors.

Then, the Actor-Partner Interdependence Model will be used to examine actor and partner effects of sexual function (model 1) and dyadic coping styles (model 2) on sexual distress of each member of the dyad [114-117]. A patient’s score on the predictor variable is expected to affect their own criterion variable scores (actor effect) and those of their partner (partner effect), and vice versa. Thus, it will be possible to determine whether sexual distress results from the score of the patients, partners, or both on the dyadic coping and sexual functioning measure [114,117,118]. Given the small sample size (60 dyads), the pooled regression approach will be used [114,117]. Stata (version 17; StataCorp) [119] will be used for the analysis, as the pooled regression approach is straightforwardly implemented using the *mvreg* command. The reasonableness of regression assumptions will be assessed using diagnostic statistics and plots [120]. Departures from assumptions will be handled using contemporary approaches such as the use of heteroscedasticity-consistent SEs. Power estimates were favorable: with a sample size of 60 dyads, assuming an α level of .05, the power of the regression *F* test to detect a significant model of each partner’s sexual function (model 1) and dyadic coping style (model 2) for sexual distress is approximately 0.86 in the presence of a medium effect size [115].

The qualitative phase of the study is designed to further explain quantitative results via purposive sampling of a subset of dyads. Interview recordings (n=20 dyadic interviews) will be transcribed verbatim and analyzed using thematic content analysis [121]. Transcripts will be reviewed for concordance, and discrepancies will be resolved through discussion among investigators. Qualitative data will be reviewed iteratively to reach saturation; that is, when no new information emerges during coding [122,123]. Atlas.ti software (ATLAS.ti Scientific Software Development GmbH) will be used to manage and organize qualitative data [124].

### Mixed Methods Integration

This study adopts an explanatory sequential mixed methods design, which incorporates quantitative and qualitative approaches as part of 2 consecutive phases [81]. Explanatory designs use qualitative data to explain and achieve greater understanding of the results emerging from the quantitative data [81,125]. In this design, the qualitative component builds on the survey and couples participating in the dyadic interview are selected from the sample that had previously completed the quantitative component. Integration of the 2 strands of data will be presented in a joint display table [126], thus yielding a more comprehensive view of participants’ experiences and illustrating interpersonal processes that inform couples’ coping with impaired sexual functioning.

## Results

Recruitment and data collection started in July 2022, and data collection is expected to be concluded in September 2024. No results are available as of manuscript preparation.

## Discussion

This mixed methods study, to the best of our knowledge, will be among the first to apply a relational perspective to the investigation of sexual health outcomes among adolescent and young adult CRC survivors. Conducting research with dyads presents numerous challenges in research design, measurement, and data analysis [96,109-111]. Approaches to recruitment and data collection have been selected on the basis of previous acceptability in similar studies conducted by members of the investigative team (CA and DW) and the existing literature on couple-based research [111-115]. In addition, age-tailored web-based data collection efforts will contribute to the study’s feasibility. We consider it a strength of the proposed study that we will integrate survey and qualitative feedback from both partners and from diverse typologies of dyads.

Addressing changes in sexual function and their implications for psychosocial and clinical outcomes is essential to quality cancer care; yet, it is often challenging to translate this knowledge into routine practices inclusive of cosurvivors [20,57]. The expected outcomes of this investigation are to (1) characterize sexual function, sexual distress, and dyadic coping of adolescent and young adult CRC survivors and partners through validated measures; (2) provide a deeper understanding of the challenges couples experience as they navigate sexual intimacy after CRC treatment by highlighting the role of interpersonal processes; and (3) use the findings to develop and test interventions supporting the recovery of sexual intimacy of young couples coping with CRC. Sexual dysfunction and altered intimacy with a partner are salient factors impairing long-term quality of life and emotional well-being [20,69,116]. Through the identification of individual, partner, and relationship-level factors that shape how couples negotiate the loss elicited by the illness and redefine their sexual experience or repertoire, this work will inform the development of a couple-based intervention that promotes intimacy by enhancing coping and communication skills. The results from this study will also contribute to identifying couples who are more vulnerable to poor emotional and relationship functioning, either because of clinical, sociodemographic, or interaction effects. This information can be used to alert the multidisciplinary team and offer timely referral to services.

While the cross-sectional design and the small sample size are limitations of this protocol, this work can lead to further interesting areas of inquiry. Future research will be needed to evaluate sexual health outcomes over time and elicit information about preferred timing, format, and contents of sexual health programs. In addition, investigators can consider integrating momentary ecological assessment approaches and dyadic diaries to better capture antecedents and patterns associated with differential trajectories. Finally, future studies should investigate whether delivering sexual health interventions results in improved quality of life and other psychosocial health outcomes among adolescents and young adults.

In conclusion, sexual health problems are among the most distressing consequences of the disease for young patients with CRC, with recent literature documenting the lack of evidence-based approaches tailored to their experience, preferences, and needs [12,20,55,127]. This study seeks to fill this gap by involving survivors and their partners to generate a dyadic model of sexual functioning and intimacy. In addition, while the literature has often linked addressing sexual health concerns with a higher quality of life, emotional well-being, and relationship functioning [67], these relationships have been minimally examined in this group. This lack of knowledge impairs the range and quality of services, especially considering the impact of sexual dysfunction, body image concerns, and fertility status on physical and emotional intimacy. As growing evidence has documented how the complexity of sexual health issues may hinder providers’ capacity and confidence to discuss this topic [127-130], results from this work will provide important recommendations for timely and patient-centered care delivery.

## Abbreviations

CRC: colorectal cancer
PROMIS: Patient-Reported Outcomes Measurement Information System

## Data Availability

The data that support the findings of this study will be available from the corresponding author (CA) upon reasonable request. The data are not publicly available due to information that could compromise the privacy of research participants.
